# Genito-Urinary and Syphilitic Diseases

**Published:** 1897-12

**Authors:** Byron H. Daggett

**Affiliations:** Buffalo, N. Y.


### GENITO-URINARY AND SYPHILITIC DISEASES.

Conducted by BYRON H. DAGGETT, M. D., Buffalo, N. Y.

### ANALYSIS OF THE URINE IN NEPHRITIS.

BIERWIRTH, M. D., (*Brooklyn Medical Journal*, November, 1897,) says diseases of the kidney are diagnosticated by the analysis of the urine and the specimen should invariably be taken from the entire output for twenty-four hours. No opinion should be based upon a specimen passed at any time of the day or night.

The entire production for twenty-four hours should be obtained to make sure of accurate measurement and the patient instructed as to the mode of collection. The urine should be passed into one or two clean bottles, corked and kept cool. The beginning of fermentation renders it unfit for analysis. To prevent fermentation, a drachm of chloral in solution should be put into the bottle, which should be well shaken after each addition. The chloral will prevent the development of bacteria and the urine will keep at all temperatures without fermentation. Alkaline fermentation destroys the microscopical elements of importance.

We cannot differentiate the various forms of nephritis by analysis of the urine, so that it is only practical to consider acute and chronic congestion and acute and chronic nephritis, and even then with the greatest care mistakes are made because we find the same urinary characteristics in the different forms of chronic inflammation of the kidney.

To make an examination of urine, note quantity, reaction, color, specific gravity, phosphates and chlorids, albumin, urea and sediment.

*Quantity.*—The average physiological quantity for twenty-four hours is about fifty ounces. The amount of urine is markedly diminished in all forms of acute nephritis, in acute exacerbations of chronic nephritis and in congestion of the kidney. There is less diminution in chronic diffuse nephritis. The urine is increased in waxy degeneration and slow forms of chronic nephritis.

*Reaction.*—In health the twenty-four-hour output is acid. It is also acid in the majority of kidney inflammations. Pus in the urine makes it faintly acid, neutral or alkaline when voided and causes it to become rapidly alkaline on standing.

*Color.*—In all acute cases the blood is usually smoky, brown or chocolate color, due to the blood in the presence of acid. If the urine is alkaline the blood imparts a red color. In chronic nephritis the urine is usually straw color and clear, and this is especially so in amyloid degeneration. When the urine becomes scanty it is a dark amber color.

*Specific gravity.*—In acute inflammation the specific gravity is high, 1030 or over, due to concentration and the presence of blood. In chronic nephritis the specific gravity is below the normal and does not increase with diminution of the amount of the urine. In the cirrhotic kidney the specific gravity is usually between 1010 to 1012. A persistent low specific gravity points to chronic nephritis, unless there be high blood pressure from lesion of the circulatory apparatus, which will increase the specific gravity.

*Phosphates* and *Chlorids* may be considered together. They give no significance, except as a sequence of indigestion. They are relatively increased in acute nephritis, due to the concentration of the urine. They are diminished in all chronic cases. In chronic nephritis, occasionally, the specific gravity is increased by the phosphates and chlorids and is not due to the increase of urea. This should be borne in mind in making a rough estimate of the amount of urea from the specific gravity.

*Albumin.*—The test for albumin is most important for diagnosis. Numerous tests have been proposed and much has been written on this subject. Blood-serum transudes from the capillaries into the tubules as a result of changes in the capillary walls; changes in the composition of blood or blood pressure. These different causes should be borne in mind, as they have a clinical and therapeutic significance.

Albumin in the urine does not always signify kidney lesion; it is found in anemia, chlorosis and eclampsia, as well as in other cases in which there is no evidence of nephritis.

Dr. Delafield recognises four varieties of albuminuria without nephritis:

*Paroxysmal cyclic albuminuria.*—It occurs in young males in poor health. There is a regular rise and fall in the amount of albumin. It appears in the morning and disappears at night. This regular cycle follows changing the hours of rest, of meals and of exercise. The cause is unknown; it may be due to the blood or the renal capillaries. The differentiation between cyclic albuminuria and nephritis is very difficult.

*Diatetic Albuminuria.*—This occurs in both children and adults and follows the ingestion of certain kinds of food. The quantity of albumin is small and few or no casts. This form is serious only if persistent.

*Albuminuria after Exertion.*—This form is due to violent and prolonged exertion, probably causing renal congestion. The albumin disappears after a few hours' or days' rest.

*Single Persistent Albuminuria.*—The albumin is not abundant, but appears nearly every day. A few hyalin casts are found. Such cases, in time, are apt to produce chronic nephritis, endocarditis or endo-arteritis.

*Tests for Albumin.*—The tests for albumin are numerous and many of them objectionable, because they precipitate both the serum albumin and the nucleo-albumin (mucin).

The contact method is objectionable, because the zone of opacity is faint and heat often disturbs it, making the result doubtful. Dr. Bierwirth employs two tests for albumin, the heat and nitric acid and the ferro-cyanid of potassium. The urine should be perfectly clear, and if not so it should be filtered through a double wet filter. It should be acid in reaction. Boil the urine in a test-tube and add 15 to 20 drops of strong nitric acid. Instant turbidity will show an appreciable quantity of albumin; if there is only a trace, turbidity will gradually appear after five or ten minutes.

Cork the tube and set it aside for six to twelve hours and usually a precipitate forms. This precipitate will be, (1) acid urates; (2) uric acid; (3) nitrate of urea; (4) albumin. The first three are redissolved by heat. Boil the second time and turbidity means albumin. This test avoids the mucin reaction, as strong acid dissolves the mucin.

The ferro-cyanid test is described in detail in Purdy's Practical Analysis; it avoids the mucin reaction, is reliable and delicate.

Albumin is found in abundance in acute congestion, waxy degeneration and in chronic congestion, with high blood pressure.

In chronic nephritis and so-called cirrhotic kidney the amount of albumin is small and absent at times.

The next most important ingredient is urea. In chronic nephritis the urine should be frequently examined, for the treatment depends upon the amount of urea eliminated. It is the associates of urea that are so pernicious in uremia.

The Doremus apparatus for estimating the amount of urea is simple and quickly used, therefore preferable. The daily amount of urea is diminished in all kidney diseases.

The tolerance of urea is variable. Urinary sediment is the most important for diagnosis; it contains all the pathological elements resulting from renal disease.

---

### GENERAL PATHOLOGY OF NEPHRITIS.

J. M. Van Cott (*Brooklyn Med. Jour.*, November, 1897,) says the kidney structure is well adapted to the study of the principles of pathology, because all the component elements of tissue structure are found therein; it is so highly endowed with function and the nature of its function is so apparent that diseased processes readily indicate structural changes.

All tissues are composed of two elements : the first is called the parenchyma and is composed of cells, which manifest an active function ; the other is intercellular substance, or stroma, and has a passive function. If the normal ratio in point of quantity of parenchyma and stroma be disturbed, the function is also disturbed. In the make-up of a complex organ these elements vary in form and function in its different portions, but its total activity will equal the sum of its various special functions during its physiological activity. In addition to parenchyma and stroma all organs are endowed with vessels, lymphatics and nerves, arranged in a systematic way and often in characteristic order of the particular organs. Organic lesions may be similar in different organs and their clinical manifestations be widely different.

Pathological changes are found in either the parenchyma, stroma or both, of organs and the primary seat may be in the vascular, nervous or lymphatic structures. The differences in the pathology of organs is due to the variety, form and function of their component elements. The parenchyma is most abundant in the cortex, and here the uriniferous tubules are lined with several varieties of epithelium, indicating a complex function. Strictly speaking the parenchyma is located in the uriniferous tubules and it is estimated that there are several miles of these tubes in the kidney. The stroma is most abundant in the medulla, which holds straight tubes lined with epithelium whose function is obscure. There are two parenchymatous functions, osmotic and secretory. The vascular apparatus presents a terminal circulation in character ; the intertubular arteries run up into the cortex without collateral circulation. The straight vessels run between the ducts of Bellini without osculation. The capillary loops in Bowman's capsule, fed by the vasa afferentia and drained by the vasa efferentia, promote osmoses of water, which dissolves the salts excreted by the epithelia of the convoluted tubules, forming urine. The lymphatics closely envelop the uriniferous tubules, thus exposing the organ to infection by pathogenic germs. The ultimate relation of the renal nerves is not known, but they are intimately associated with the individual tubules, indicating that the trophic fibers enter the parenchymatous elements.

Renal lesions are parenchymatous, stromatous or diffuse and are caused by changes in the quality and quantity of the blood and lymph ; by trophic changes in the ultimate parenchymatous elements and primary cardio-vascular lesions. Nephritis may be acute or chronic and involve one or all of the elementary structures. Acute nephritis is parenchymatous or diffuse.

In acute nephritis there is rapid epithelial degeneration, hemorrhage into the tubules, epithelial desquamation and albuminous exudation. Long-continued toxemias induce parenchymatous changes and hyperplasia of stroma. Chronic hyperemia results in chronic hyperplasia of connective tissue and ultimate destruction of the parenchyma. Whether parenchymatous atrophy is a result of chronic interstitial hyperplasia, or whether it occurs *pari passu* with the change in the stroma, is a mooted question. The result will be atrophy with formation of cysts in the cortex and contraction of the uriniferous tufts and obliteration of the Malphigian tuft. The epithelium atrophies and undergoes fatty degeneration, with pigmentation from long-standing hemorrhage.

Changes in the kidney simulate changes in the liver and may be regarded as chronic parenchymatous degeneration, hypertrophic or atrophic cirrhosis and disturbances due to vascular lesions. Acute parenchymatous nephritis is due to acute renal hyperemia; acute diffused nephritis results from septic infection. Chronic renal lesions are resultant from various causes.

The primary vascular lesions are: (1) White infarct; (2) hemorrhagic infarct; (3) septic thrombosis and embolism; (4) endarteritis obliterans.

White infarct is due to oligemia, red infarct to hyperemia, venous stasis and hemorrhage, and both result in coagulation, necrosis, diffuse atrophy and cicatrisation.

Microbic infection causes lymphatic lesions, since the lymph channels are the common carriers of germs, and the particular kind of germ decides the ultimate pathological lesion. Certain cardiac lesions produce chronic renal hyperemia and characteristic renal changes and characteristic changes in the urine.

Diseases of the kidneys are no more complicated than those of the other organs; any cause that will produce lesion of the kidneys is calculated to produce disease in the other organs, whether acute or chronic. Lesions of the glomerular epithelium result in albuminuria; lesions of the tubular epithelium result in reduction of elimination of urea and other salts.

---

### HYDROZONE IN DISORDERS OF THE GENITO-URINARY TRACT.

Dr. John Aulde (*Medical Times and Register*,) states that about eight years ago he was impressed with the value of peroxide of hydrogen in a protracted case of gonorrhea. The disease had persisted for three months despite treatment, there being a constant discharge and in addition an orchitis was present, the left testicle being about as large as a baseball. Treatment: injections of equal parts of peroxide of hydrogen and moderately warm water at intervals of four hours, the injections being followed by a solution of arsenite of copper, one milligram (one 65th grain) to the drachm, diluted with an equal quantity of hot water.

In a week the discharge from the urethra entirely ceased and pain and chordee disappeared.

Aulde advises the same treatment for nonspecific urethritis and gleet, but as hydrozone is much stronger (2 times) than the peroxide and perfectly harmless he gives it the preference.

In vaginitis and vaginismus this treatment is of especial value. The treatment heretofore recommended by physicians, consisting of hot vaginal douches, either with or without some alkali, as sodium bicarbonate, followed by the injection of a small quantity of peroxide of hydrogen (medicinal) in warm or cold water, is superseded by the single application of a hot solution of hydrozone, one part in eight; the patient should use a fountain syringe hung upon the wall six feet from the floor. The injection should be repeated twice in twenty-four hours.

In uterine diseases, where the solution must be brought into contact with the endometrium, the following treatment is pursued: The patient is placed in the dorsal position, with the hips well elevated; an ordinary dilator is employed to distend the cervix, so as to admit the nozzle of the syringe and permit the free egress of the injected fluid; (a suitable return flow tube can be used to better advantage—the Fritsche's douche is the best that can be used). The injection is then made, a liberal amount of the hot medicated solution being used.

There is need of caution in chronic cases that the effervescence which attends the destruction of the unhealthy tissue does not force some of the débris into the Fallopian tubes. This is best avoided by using a large quantity of the solution and afterward directing the patient to assume the upright position. The pressure thus brought to bear upon the uterus will cause the complete discharge of all débris.

A preliminary vaginal douche should always be taken, using the medicated solution, as otherwise harm might ensue by the entrance into the uterus of the vaginal secretions. The author warns against the use of the vaginal douche if the cervix be patulous, as there is an almost certain danger of the infected vaginal débris being forced into the uterine cavity. To avoid this the vagina should be cleansed by the local use of the medicated solution through the speculum.

The author believes hydrozone to be the best remedy for cystitis occurring either in the male or female. The bladder should be washed out with the solution (one to eight), a small quantity being used at first in chronic cases, owing to the painful muscular contractions following the withdrawal of the solution. The amount can be gradually increased. (A double current hard rubber catheter should always be used for that purpose). In gonorrhea, gleet and cystitis, the local treatment is oftentimes aided by the internal administration of hourly doses of calcium sulphide, one-tenth of a grain.

